# Instant multicolor super-resolution microscopy with deep convolutional neural network

**DOI:** 10.52601/bpr.2021.210017

**Published:** 2021-08-31

**Authors:** Songyue Wang, Chang Qiao, Amin Jiang, Di Li, Dong Li

**Affiliations:** 1 National Laboratory of Biomacromolecules, CAS Center for Excellence in Biomacromolecules, Institute of Biophysics, Chinese Academy of Sciences, Beijing 100101, China; 2 College of Life Sciences, University of Chinese Academy of Sciences, Beijing 100049, China; 3 Department of Automation, Tsinghua University, Beijing 100084, China; 4 Hefei National Laboratory for Physical Sciences at Microscale and School of Life Sciences, University of Science and Technology of China, Hefei 230026, China; 5 Bioland Laboratory (Guangzhou Regenerative Medicine and Health Guangdong Laboratory), Guangzhou 510005, China

**Keywords:** Multicolor imaging, Super-resolution, Convolutional neural network

## Abstract

Multicolor super-resolution (SR) microscopy plays a critical role in cell biology research and can visualize the interactions between different organelles and the cytoskeleton within a single cell. However, more color channels bring about a heavier budget for imaging and sample preparation, and the use of fluorescent dyes of higher emission wavelengths leads to a worse spatial resolution. Recently, deep convolutional neural networks (CNNs) have shown a compelling capability in cell segmentation, super-resolution reconstruction, image restoration, and many other aspects. Taking advantage of CNN’s strong representational ability, we devised a deep CNN-based instant multicolor super-resolution imaging method termed IMC-SR and demonstrated that it could be used to separate different biological components labeled with the same fluorophore, and generate multicolor images from a single super-resolution image *in silico*. By IMC-SR, we achieved fast three-color live-cell super-resolution imaging with ~100 nm resolution over a long temporal duration, revealing the complicated interactions between multiple organelles and the cytoskeleton in a single COS-7 cell.

## INTRODUCTION

There are countless complicated yet well-regulated interactions between the organelles and the cytoskeleton in every cell, which are aimed at maintaining cellular homeostasis and executing various physiological functions (Phillips and Voeltz 2016; Prinz 2014). Observing the intracellular dynamics of multiple organelles and the cytoskeleton is of vital importance for cell biology. Unfortunately, there are still several barriers for long-term multicolor and high spatial-temporal resolution live-cell imaging, which mainly lie in the following two aspects: the limited spatial resolution by optical diffraction theory and the negative effects created by excitation lasers, *i*.*e*., photobleaching and phototoxicity. To break through the optical diffraction limits, several super-resolution methods have been proposed, *e*.*g*., structured illumination microscopy (SIM) (Gustafsson *et al*. 2008), stimulated emission depletion microscopy (STED) (Willig *et al*. 2006), photoactivated localization microscopy (PALM) (Shroff *et al*. 2008) and stochastic optical reconstruction microscopy (STORM) (Rust *et al*. 2006). However, although superior spatial resolution is achieved, these SR methods suffer from respective defects, *i*.*e*., repetitive acquisitions for SIM, heavy photon budget for STED, and low temporal resolution for PALM and STORM. Furthermore, when performing multicolor imaging, one region of interest (ROI) will be illuminated two or more times for each time point, thus causing heavier degradation of cytoactivity and restricting the maximal frame rate, which distinctly limits the widespread adoption of various SR methods in multicolor live-cell imaging experiments.

In recent years, deep learning has become an increasingly popular approach to be applied in multiple biomedical imaging processing or analysis tasks, *i*.*e*., extracting quantitative information from biological images (Isensee *et al*. 2021; Kermany *et al*. 2018; Moen *et al*. 2019; Pan *et al*. 2019), performing restoration or cross-modality transformation for microscopic images (Wang *et al*. 2019; Weigert *et al*. 2018) and reconstructing super-resolution images from a reduced number of corrupted raw data (Qiao *et al*. 2021b). In particular, with the help of elaborate convolutional neural network models such as U-net (Ronneberger *et al*. 2015), researchers can perform cell or organelle segmentation with high accuracy and efficiency (Isensee *et al*. 2021; Ronneberger *et al*. 2015), indicating that deep convolutional neural networks have a significant capability for image feature extraction and representation. Inspired by this, we made a step forward in using a deep CNN model to directly separate various subcellular structures from a single SR image, rather than only performing segmentation as others, and then recombining them again, which is equivalent to an instant multicolor SR (IMC-SR) procedure. The major benefits of this multicolor imaging framework are threefold: first, all of the biological structures can be labeled in the same fluorescent channel of relatively short wavelength, *i*.*e*., the green channel, thus simplifying the sample preparation and potentially enhancing the spatial resolution; second, the IMC-SRscheme avoids repetitive exposure and acquisitions for each channel, which can relieve photobleaching and phototoxicity; third, the contents of each channel are captured simultaneously, thereby avoiding misinterpretations due to conventional sequential multicolor acquisitions. Furthermore, considering that U-net is a kind of wide but shallow neural network model, and it has been found that deeper CNN models may lead to a better performance than shallow models, especially when dealing with computationally complicated tasks (Lim *et al*. 2017), we adopted a modified deep CNN model based on residual learning (Szegedy *et al*. 2017) and a channel attention mechanism (Hu *et al*. 2018; Zhang *et al*. 2018) to perform multicolor image separation.

In this work, we first presented the conception and workflow of our IMC-SR method and explored and optimized the application strategies of our deep CNN model. Then, we performed statistical evaluation according to the application strategies, different types of subcellular structures and input signal-to-noise-ratio (SNR) conditions. Finally, we demonstrated its effectiveness in experimental usage by observing three different types of intracellular structures simultaneously in a single COS-7 cell of ~100 nm resolution over a long temporal duration, visualizing the complicated interactions between different organelles and cytoskeleton.

## RESULTS

### Network architecture

Our IMC-SR neural network separates components of different types of biological structures from a single monochrome grayscale SR image in which all the information is superimposed. Compared with cell or organelle segmentation tasks, the separation of different biological components is a more complicated and underdetermined problem because separation means the network needs to determine the intensity values of every pixel for each output channel instead of only providing a binary mask in segmentation tasks. Furthermore, SR images contain many subtler details that are finer than the diffraction limits, thus increasing the structure separation difficulty. Therefore, we constructed our CNN model following two criteria: first, the network model should be deep enough to be competent in informative feature extraction and representation; second, the network model should be trainable and computationally efficient. Inspired by the SOTA single image super-resolution architecture residual channel attention network (RCAN) (Zhang *et al*. 2018), we devised a shallower version of RCAN, especially for multicolor image separation (hereafter named the IMC-SR network), which exhibits higher robustness and efficiency than the RCAN baseline model in our tasks.

Our IMC-SR network begins with a conv-GELU module for shallow feature extraction, which consists of one 3 × 3 convolutional layer and a GELU activation layer (Hendrycks and Gimpel 2016). The output of the conv-GELU module is fed into five consecutive residual groups (RG). Each RG is composed of ten residual channel attention blocks (RCABs) and a skip connection pointing from the RG input to the output of the last RCAB. RCAB is the core component of the IMC-SR network, which consists of two conv-GELU modules and a channel attention module. In channel attention modules, each feature channel is adaptively rescaled according to the interdependency of all the feature channels, thus rendering the network focused on more meritorious information (Zhang *et al*. 2018). The output of the last RG is followed by an extra conv-GELU module to reorganize the feature channels, and a convolutional layer with sigmoid activation is used to generate the final monochrome image containing specific types of biological structure.

### Data acquisition and preprocessing

Training and testing datasets of the endoplasmic reticulum (ER), microtubules (MTs) and lysosomes (Lyso) were acquired using our home-built multimodality structured illumination microscopy (TIRF-SIM and GI-SIM) (Guo *et al*. 2018; Li *et al*. 2015). For each type of specimen, we acquired 30 sets of raw SIM images (512 $ \times $ 512) of different regions of interest (ROIs) and reconstructed them into 30 SR-SIM images (1024 $ \times $ 1024) using a conventional SIM reconstruction algorithm (Gustafsson *et al*. 2008; Li *et al*. 2015). Each SR-SIM image was then augmented into 50 image patches of 128 $ \times $ 128 pixels by flipping, random cropping and random rotation, constituting three sets of image patches corresponding to three types of biological specimens. To obtain paired data for training and testing, we randomly selected one image patch from each patch set separately and added them together as the input to the IMC-SR network. Correspondingly, the three original SR image patches serve as the ground truth (GT) in the training process.

### Network training and evaluation

To evaluate the effectiveness of our IMC-SR method, we trained three individual IMC-SR network models for separating ER, MTs and Lyso from a single superimposed image of these three subcellular structures. The objective function we used in the training process was the combination of mean square error (MSE, *M*) and structural similarity (SSIM, *S*) as follows:

1 $ {{L}}\left(\widehat{y},y\right)=\lambda \cdot {{M}}\left(\widehat{y},y\right)+\mu \cdot {{S}}\left(\widehat{y},y\right), $

where “$ \lambda $” and “$ \mu $” are scalar weighting factors set as 1 and 0.1 in our experiments, respectively, “$ \widehat{y} $” represents the output image of the network, and “$ y $” is the ground-truth image.

**Figure 1:**
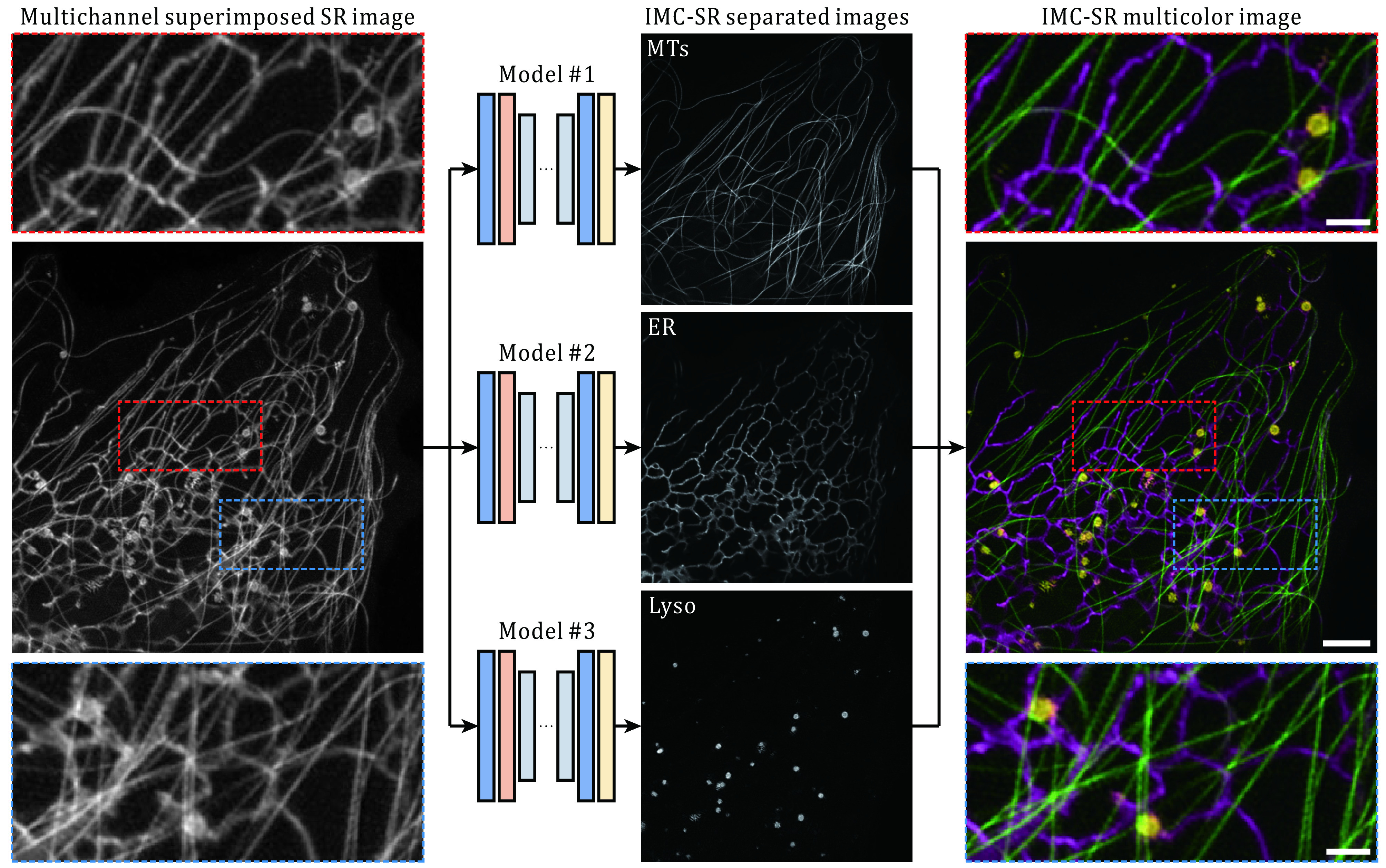
Schematic and representative results of IMC-SR methods

**Figure 2:**
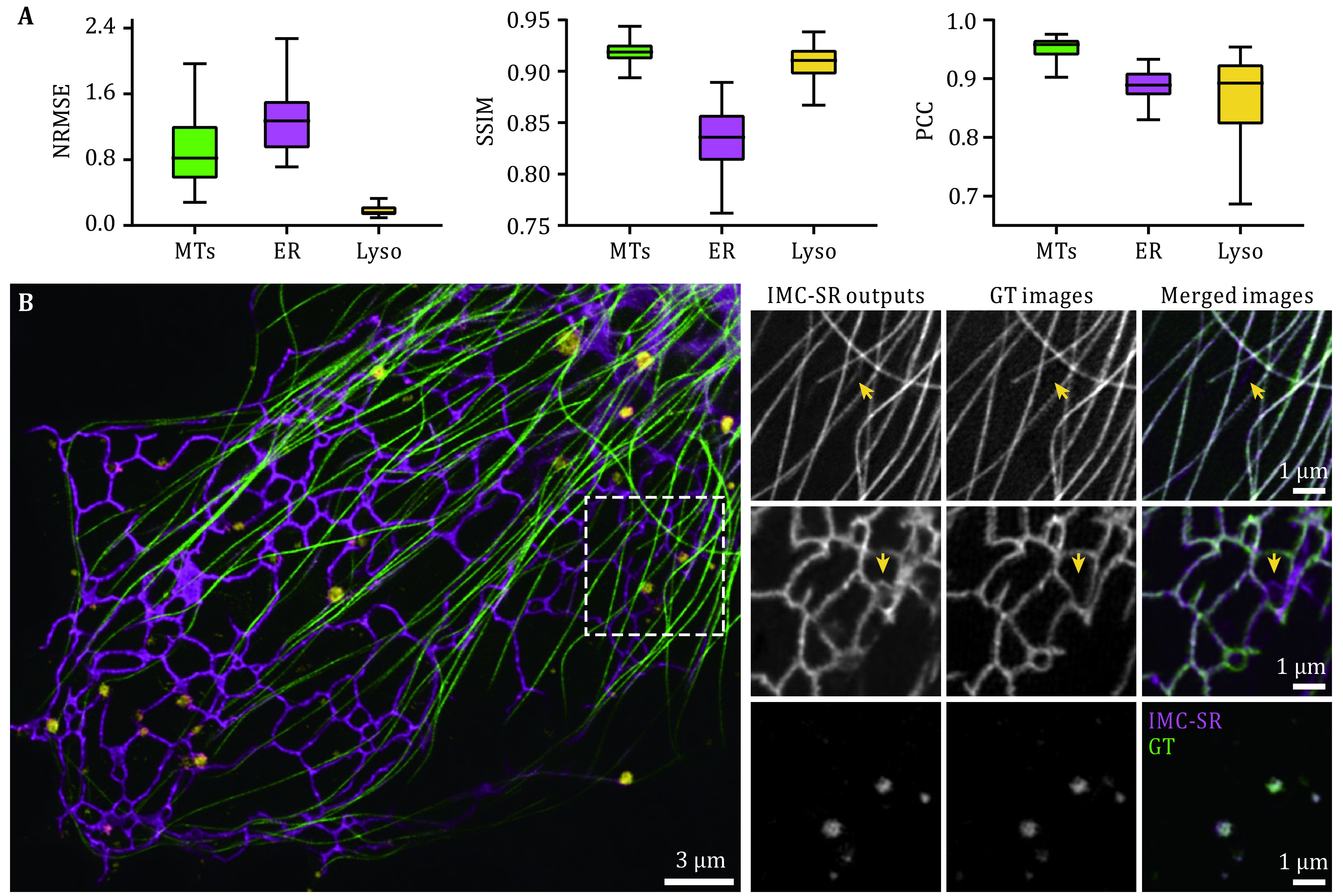
Evaluation and representative results of IMC-SR-Single models. **A** Statistical analysis of IMC-SR-Single models trained for MT, ER and Lyso in terms of NRMSE, SSIM and PCC. **B** Representative results of IMC-SR-Single models trained for MT (green), ER (magenta) and Lyso (yellow). Yellow arrows indicate the false-positive errors of network output images. Scale bar: 3 μm and 1 μm (zoom-in regions)

After being well trained, the three IMC-SR models were applied to the same input SR image containing both ER, MTs and Lyso. It can be clearly seen that our IMC-SR network models successfully separated the tubular structure of MTs, the reticulate structure of ER and the punctate structure of Lyso, and these details were observed together in an integrated multicolor image (Fig. 1). To statistically evaluate the performance of IMC-SR methods, we calculated the normalized root mean square error (NRMSE), SSIM and Pearson correlation coefficient (PCC) on 100 output images and 100 corresponding GT images for each model (Fig. 2A). The upper and lower whiskers of the Tukey box-and-whisker plots show the performance boundary of each model, and the median values (lines in the middle of the boxes) indicate that IMC-SR methods achieve fairly good multicolor separation quality. Although the output images of IMC-SR methods were perceptually satisfactory in general, there did exist, however, some noticeable false-positive errors in the separated images (yellow arrows in Fig. 2B).

### Combination loss to alleviate false-positive errors

After carefully comparing the outputs of IMC-SR modes with the GT images, we found that for a certain model, false-positive errors are actually the misrecognition of contents from other color channels. To alleviate them, we explored a new training strategy, that is, training a sole model directly outputting all channels of biological structure (hereafter termed IMC-SR-All) instead of training models for every single type of structure (hereafter termed IMC-SR-Single). The most conspicuous benefit of this strategy is that we also utilized the original input image to supervise the iterative updating during the training procedure (hereafter termed summation loss) with the objective function formulated as

2 \begin{equation*}\begin{split} 
{{L}}\left(x,\widehat{y},y\right)=&\lambda \cdot {{M}}\left(\widehat{y},y\right)+\mu \cdot {{S}}\left(\widehat{y},y\right)\\&
+\alpha \{\lambda \cdot {{M}}[sum\left(\widehat{y}\right),x]+\mu \cdot {{S}}[sum\left(\widehat{y}\right),x\left]\right\},
\end{split}\end{equation*}

where “$ sum\left( \cdot \right) $” indicates the channelwise summation operator, “$ x $” is the input image of the IMC-SR-All model and “$ \alpha $” is another weighting scalar to balance the summation loss, which is empirically set as 0.02 in our experiments.

**Figure 3:**
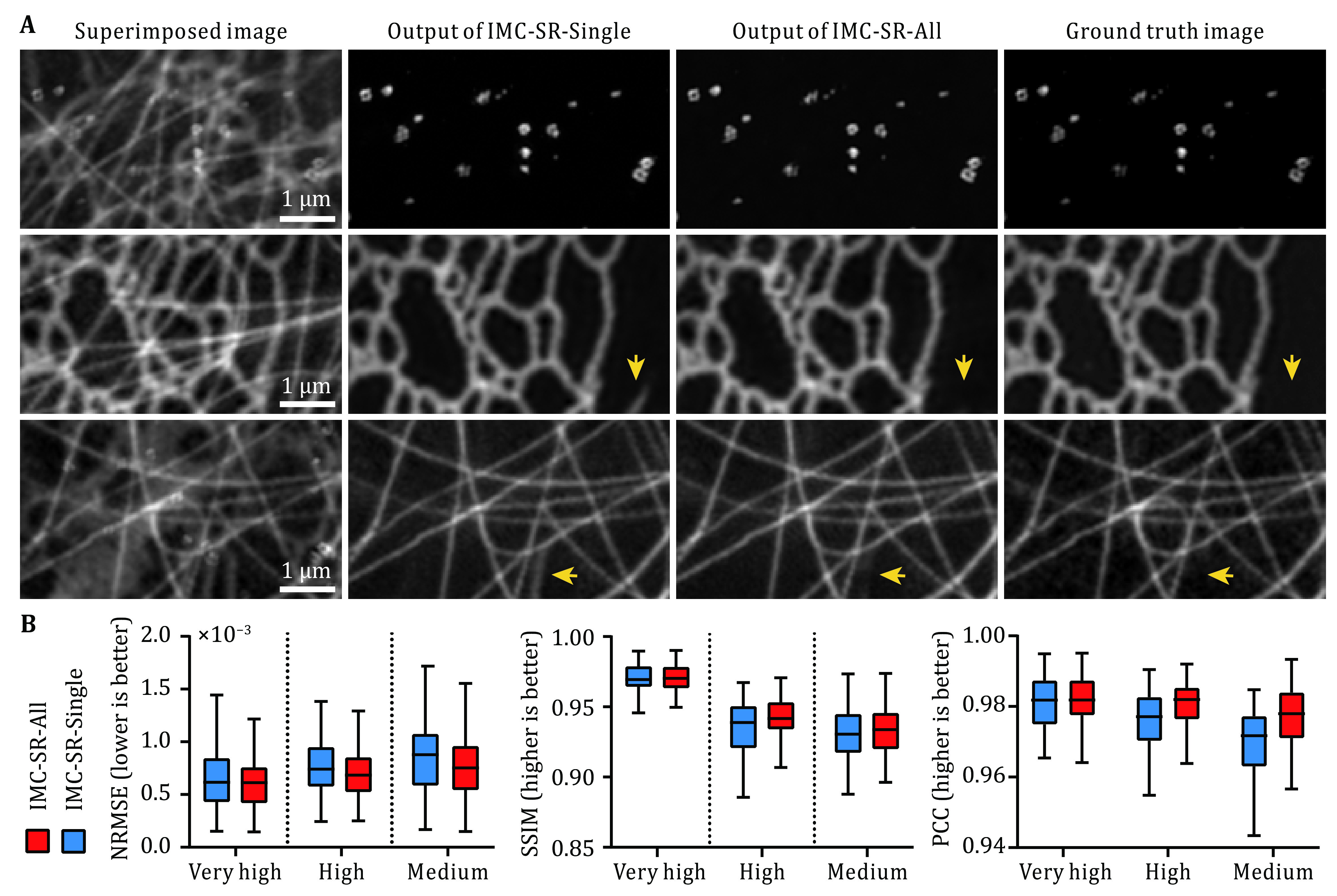
Comparisons of IMC-SR-All models and IMC-SR-Single models. **A** Representative results of the IMC-SR-All model and IMC-SR-Single models trained for CCPs, ER and MTs. Arrows show that the false-positive errors can be reduced considerably with combination loss. Scale bar: 1 μm. **B** Statistical analysis of the IMC-SR-All model and IMC-SR-Single models trained for ER in terms of NRMSE, SSIM and PCC at different input SNR conditions

To evaluate the practicability and robustness of IMC-SR-All under different input SNR conditions, we trained one IMC-SR-All model for clathrin-coated pits (CCPs), MTs and ER using the data from our previously published dataset BioSR (Qiao *et al*. 2021b) following the training procedure described above. It is noteworthy that we trained only one IMC-SR-All model for separating all subcellular structures and SNR conditions, which covered medium SNR (relatively low quality with slight artifacts after SIM reconstruction), high SNR (most common imaging conditions in daily experiments) and very high SNR (superior quality without reconstruction artifacts). We also trained three IMC-SR-Single models for CCPs, MTs and ER individually for comparison. The results shown in Fig. 3A indicate that with the regularization of the combination loss described in Eq. 2, the false-positive errors were reduced considerably (arrows in Fig. 3A), and the statistical analysis in terms of NRMSE, SSIM and PCC in Fig. 3B shows that the IMC-SR-All model achieved better performance than IMC-SR-Single models in all SNR conditions.

### Enhancing performance of IMC-SR for time-lapse data by virtue of temporal continuity

**Figure 4:**
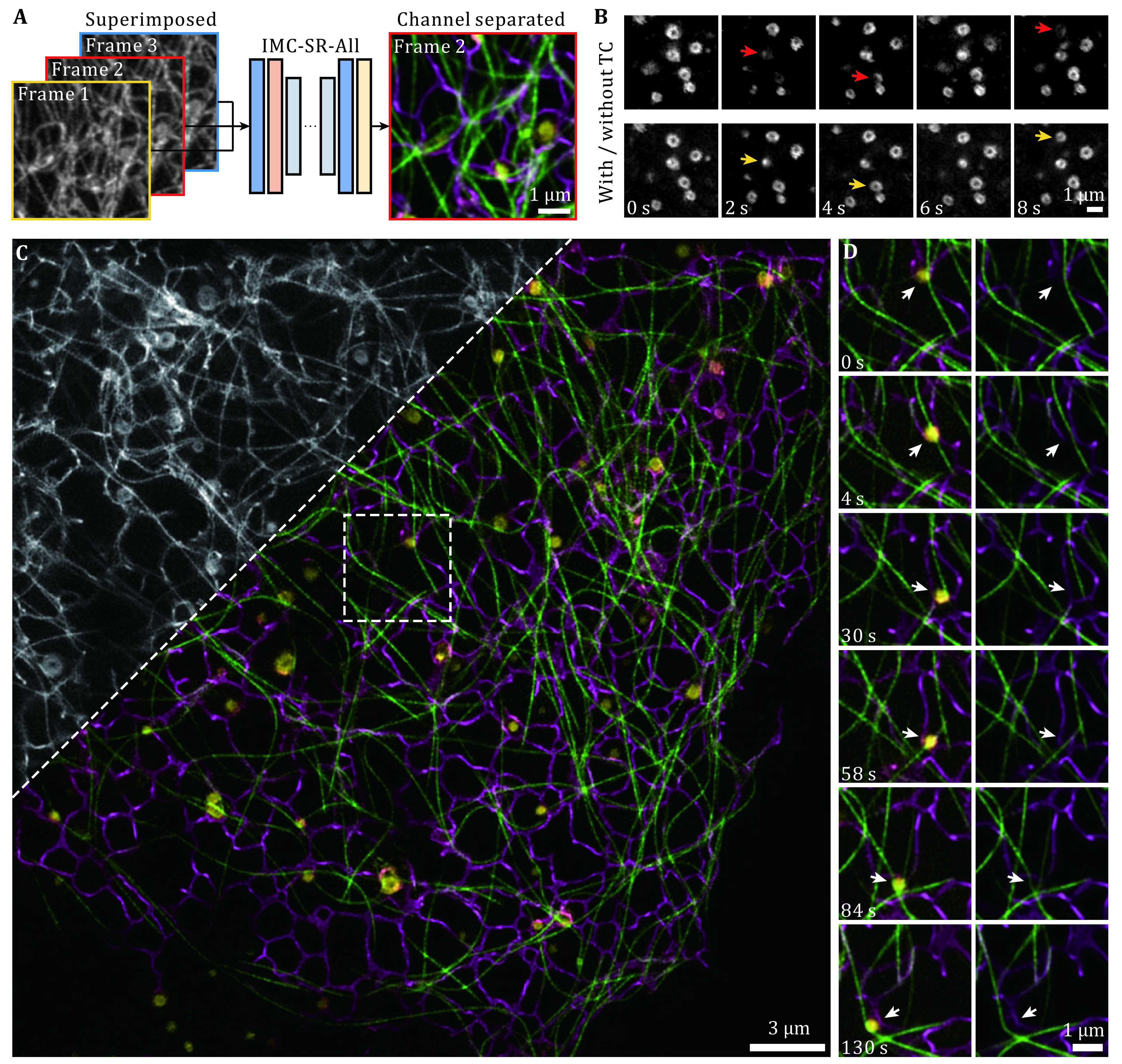
Instant three-color live-cell SR imaging by IMC-SR. **A** Schematic of IMC-SR-All models applied with temporal continuity (TC). **B** Comparisons of IMC-SR-All models applied with (lower row) or without (upper row) temporal continuity. **C** Representative three-color images of MT (green), ER (magenta) and Lyso (yellow) generated by the IMC-SR-All model. Top left: a fraction of the corresponding multichannel-superimposed input image. **D** Time-lapse three-color images showing a lysosome moving along MTs that continuously contacted the ER and the hitchhiking remodeling mechanism of the ER. Gamma value: 0.8 for MT channel (**C** and **D**). Scale Bar: 1 μm (**A**, **B** and **D**), 3 μm (**C**)

We next determined that IMC-SR-All network models, trained solely on static images, can be used to separate the intracellular structure of time-lapse data, enabling instant multicolor live-cell imaging. To further enhance the performance of IMC-SR methods when dealing with time-lapse data, we harnessed temporal continuity (TC) to conditionally instruct the training and inference progress of network models. For each training or inference trial, IMC-SR models were fed with three multichannel-superimposed frames of sequential timepoints but only performed channel separation for the median timepoint (Fig. 4A). Resorting to this scheme, IMC-SR models resolved the movements and interactions of organelles and cytoskeleton more stably along the temporal dimension without undesired blinks (arrows in Fig. 4B). We demonstrated that our IMC-SR method achieved instant three-color SR imaging by visualizing the delicate interaction dynamics of MT, ER and Lyso in COS-7 cells (Fig. 4C, supplementary Video 1). One time-lapse example shows that a lysosome moves along MTs and continuously contacts the ER, concomitantly changing the morphological distribution of the ER (Fig. 4D) and verifying its hitchhiking remodeling mechanism (Guo *et al*. 2018).

## DISCUSSION

In this work, we introduced IMC-SR, a deep CNN-based analytical method, to perform channel separation directly from a single multistructure superimposed SR image, enabling fast multicolor super-resolution live-cell imaging. To improve the performance of IMC-SR models, we devised the combination loss that leverages the original input grayscale but informative images to regularize the training process of the model, thus effectively reducing the false-positive errors; to utilize the temporal continuity of time-lapse data, we conditionally provided the IMC-SR models with multiple consecutive timepoints as its input during training and inference procedures, leading to a more robust performance when handling time-lapse data in daily experiments. Finally, we applied the IMC-SR method to achieve instant three-color super-resolution live-cell imaging of subcellular structures at 2-second intervals over a duration of 100 timepoints, which reveals the sophisticated interaction between different organelles and the cytoskeleton.

Furthermore, although our IMC-SR method was only demonstrated and evaluated based on linear SIM data in this paper, apparently, it is appropriate for SR images of other imaging modalities, *e*.*g*., nonlinear SIM, STED, STORM, and PALM. Moreover, IMC-SR methods have the potential to perform channel separation and SR image restoration simultaneously if trained with artifact-corroded inputs, *i*.*e*., SIM SR images reconstructed from noise raw images and corresponding clear GT images of high quality, which could be a desirable application when there is difficulty in acquiring ideal multichannel-superimposed data for network input.

Despite the inspiring multicolor imaging performance, it is worth noting that there are some restrictions for IMC-SR methods. First, if the biological structures to be separated in the input SR image are too similar to each other, *i*.*e*., punctate CCPs and lysosomes, IMC-SR models will struggle to tell them apart. Second, in live-cell imaging experiments, the signal intensities of different biological structures are not always approximately comparable due to the susceptible expression of fluorescent protein, in which circumstance IMC-SR models cannot correctly identify certain structures if their fluorescence is too much weaker than others in a single sCMOS frame. Third, imputing to the limited generalization ability, which is the case for the majority of CNN-based methods, individual IMC-SR models should be trained for every different combination of biological structures.

Equipped with IMC-SR methods, traditional multicolor fluorescence microscopy can achieve faster multicolor imaging of high spatiotemporal resolution at prominently lower photobleaching and phototoxicity. We hope that the superior performance and extensive application potential of the IMC-SR method can shed light on new discoveries in life science research necessitating multicolor SR imaging in the future.

## METHODS

### Detailed information of network training and testing

The IMC-SR models in this paper were trained and applied on a computer workstation equipped with a Xeon Gold 6134 CPU@ 3.20 GHz (Intel) and two RTX 3090Ti graphic processing cards (NVIDIA) using Python 3.6, Tensorflow 2.4.0. In the training phase, the dataset was augmented into 20,000 image patch pairs of 128 $ \times $ 128 pixels. The initial learning rate was set to $ 1\times {10}^{-4} $, and reduced by half if the validation NRMSE doesn’t decrease for 10,000 iterations. We adopted a batch size of 3, and the training process typically took 10 hours and ~200,000 mini batch iterations. Once trained, it typically takes less than 250 milliseconds to process a multi-channel superimposed SR image of 512 $ \times $ 512 pixels for both IMC-SR-All models and IMC-SR-Single models.

### Calculation of different evaluation metrics

In this paper, we used three quantitative metrics, NRMSE, SSIM and PCC to evaluate the performance of IMC-SR models according to GT. Before calculating them, we performed the percentile normalization and linear transformation (LT) for IMC-SR model output images and GT images at first. This is a commonly used data preprocessing procedure (Qiao *et al*. 2021a; Weigert *et al*. 2018), which can be formulated as

3 $ {Y}_{N}=\frac{Y-perct(Y,{p}_{{\rm{low}}})}{perct(Y,{p}_{{\rm{high}}})-perct(Y,{p}_{{\rm{low}}})}, $

4 $ {\widehat{Y}}_{N}=\frac{\widehat{Y}-perct(\widehat{Y},{p}_{{\rm{low}}})}{perct(\widehat{Y},{p}_{{\rm{high}}})-perct(\widehat{Y},{p}_{{\rm{low}}})}, $

5 $ {\widehat{Y}}_{T}=\alpha {\widehat{Y}}_{N}+\beta, $

where “$ \widehat{Y} $”, “$ {\widehat{Y}}_{N} $”, “$ {\widehat{Y}}_{T} $”, “$ Y $”, “$ {Y}_{N} $” indicate the network output image, percentile normalized output image, linear transformed output image, GT image and percentile normalized GT image, respectively; “$ perct(Y,p) $” represents the intensity value of “$ Y $” ranking “$ p\% $” with “$ {p}_{\mathrm{l}\mathrm{o}\mathrm{w}} $” and “$ {p}_{\mathrm{h}\mathrm{i}\mathrm{g}\mathrm{h}} $” are 0.1 and 0.99 in our experiments; “$ \alpha $” and “$ \beta $” are the hyper parameters of LT, which can be determined by solving the least square problem:

6 $ \mathop {{\rm{arg}}\;{\rm{min}}}\limits_{\alpha ,\beta } {||\alpha {\widehat{Y}}_{N}+\beta -{Y}_{N}||}_{2}. $

Then the NRMSE, SSIM and PCC can be calculated as:

7 $ {\rm{NRMSE}}\left({\widehat{Y}}_{T},{Y}_{N}\right)=\sqrt{\frac{1}{w\times h}\sum \limits_{i=1}^{w\times h}{\left({\widehat{Y}}_{T, i}-{Y}_{N, i}\right)}^{2}}, $

8 $ {\rm{SSIM}}\left({\widehat{Y}}_{T},{Y}_{N}\right)=\frac{\left(2{\mu }_{{\widehat{Y}}_{T}}{\mu }_{{Y}_{N}}+{c}_{1}\right)\left({\sigma }_{{\widehat{Y}}_{T}{Y}_{N}}+{c}_{2}\right)}{\left({\mu }_{{\widehat{Y}}_{T}}^{2}+{\mu }_{{Y}_{N}}^{2}+{c}_{1}\right)\left({\sigma }_{{\widehat{Y}}_{T}}^{2}+{\sigma }_{{Y}_{N}}^{2}+{c}_{2}\right)}, $

9 $ {\rm{PCC}}\left({\widehat{Y}}_{T},{Y}_{N}\right)=\frac{{\sigma }_{{\widehat{Y}}_{T}{Y}_{N}}}{{\sigma }_{{\widehat{Y}}_{T}}{\sigma }_{{Y}_{N}}}, $

where “$ {\mu }_{X} $”, “$ {\sigma }_{X} $” and “$ {\sigma }_{XY} $” indicate the mean value of “$ X $”, standard deviation of “$ X $” and the covariation of “$ X $” and “$ Y $”; “$ {c}_{1} $” and “$ {c}_{2} $” are non-zero constants of small value used to prevent the denominator from being close to zero, which are set to 0.01 and 0.03 by default in MATLAB 2017b.

### Imaging conditions of live-cell experiments

In the three-color live-cell SR imaging experiment shown in Fig. 4, we acquired raw images in SIM mode of three-phase × three-orientation for 100 frames at 2-s intervals. Each raw image was acquired with a 488 nm illumination intensity of 40 W/cm^2^ and 30 ms exposure time for 3xmEmerald-Ensconsin, 560 nm illumination intensity of 15 W/cm^2^ and 30 ms exposure time for KDEL-mCherry, and 640 nm illumination intensity of 18 W/cm^2^ and 30 ms exposure time for LAMP1-Halo.

### Cell culture, transfection and staining

The COS-7 cell line was grown in Dulbecco’s Modified Eagle Medium (DMEM) (Gibco, Cat. No.: 11965-092) and supplemented with 10% fetal bovine serum (Gibco, Cat. No.:16140071) and 1% penicillin/streptomycin at 37 °C and 5% CO_2_ until 60%–80% confluency was reached. After coating 25-mm coverslips with 50 mg/mL collagen for 1 h, cells were seeded onto the coverslips to achieve ~70% confluence before transfection. The manufacturer’s protocol of Lipofectamine 3,000 (Invitrogen) was employed for transient transfections. The plasmid constructs used in this study include Clathrin-mEmerald, 3xmEmerald-Ensconsin, KDEL-mCherry and LAMP1-Halo.

### Statistics and reproducibility

Figures 2A and 3B were plotted in Tukey box-and-whisker format. The box plots extend from the 25th and 75th percentiles and the line in the middle of the box represents the median value. The upper whisker indicates the larger value between the largest data point and the 75th percentiles plus 1.5× the interquartile range, and the lower whisker indicates the smaller value between the smallest data point and the 25th percentiles minus 1.5× the interquartile range. Data points larger than the upper whisker or smaller than the lower whisker are identified as outliers. In the evaluation experiments in Fig. 2A and 3B, the statistics for each type of intracellular structure and SNR were calculated from 100 image pairs of 512 × 512 pixels, which were augmented following similar procedure to training phase from more than 20 different ROIs of 1024 × 1024 pixels.

## Conflict of interest

Songyue Wang, Chang Qiao, Amin Jiang, Di Li and Dong Li declare that they have no conflict of interest.
